# Subliminal determinants of cue-guided choice

**DOI:** 10.1038/s41598-020-68926-y

**Published:** 2020-07-17

**Authors:** Sara Garofalo, Laura Sagliano, Francesca Starita, Luigi Trojano, Giuseppe di Pellegrino

**Affiliations:** 1 Department of Psychology, University of Bologna, Cesena, Italy; 2 Department of Psychology, University of Campania “L. Vanvitelli”, Caserta, Italy

**Keywords:** Neuroscience, Cognitive neuroscience, Reward

## Abstract

By anticipating potential rewards, external cues can guide behavior to achieve a goal. Whether the conscious elaboration of these cues is necessary to elicit cue-guided choices is still unknown. The goal of the present study is to test whether the subliminal presentation of a visual cue previously paired with a reward is sufficient to bias responses that can lead to the same or a similar reward. To this aim, three experiments compared the subliminal and supraliminal presentation of reward-associated cues during a Pavlovian-to-Instrumental Transfer task. In line with previous evidence, results showed that the supraliminal presentation of reward-associated Pavlovian cues biased participant’s choice towards motivationally similar rewards (general transfer) as well as towards rewards sharing the precise sensory-specific properties of the cue (outcome-specific transfer). In striking contrast, subliminal cues biased choice only towards motivationally similar rewards (general transfer). Taken together, these findings suggest that cue-guided choices are modulated by the level of perceptual threshold (i.e., subliminal vs supraliminal) of reward-associated cues. Although conscious elaboration of the cue is necessary to guide choice towards a specific reward, subliminal processing is still sufficient to push towards choices sharing the motivational properties of the cue. Implications for everyday life, clinical conditions, and theoretical accounts of cue-guided choices are discussed.

## Introduction

Even when irrelevant to choice, reward-associated cues are a powerful modulator of motivated behavior^1^. In everyday life, environmental cues can anticipate the presence of potential rewards, or punishments, thus biasing choice (i.e., cue-guided choices). Although evidence shows that the mere presence of reward-associated cues can influence individual choice^2–4^, the boundary conditions within which this occurs remain unknown. In particular, it is not clear whether the conscious elaboration of such cues is necessary to elicit cue-guided choices^5,6^. However, such understanding has the potential to elucidate the mechanisms underlying maladaptive forms of cue-guided choice and potentially informing their treatment. For example, evidence points to the role of the obesogenic environment—saturated with food logos and advertisement—in the promotion of maladaptive forms of cue-guided choices, like food overconsumption^7,8^ or drug addiction^9,10^. Nevertheless, the extent to which such maladaptive choices can occur through mechanisms bypassing conscious awareness of the reward-associated cues remains unexplored.

A direct way to investigate whether the conscious elaboration of environmental cues is necessary to elicit cue-guided choices is to test whether the subliminal presentation of such cues is sufficient to influence choice. A subliminal cue is defined as any cue whose intensity is below the level of consciousness but receives perceptual and semantic processing^5,11^. In other words, a subliminal stimulus is too weak to be explicitly recognized but is still able to activate the sensitive afferent pathways^12^.

**Figure 1:**
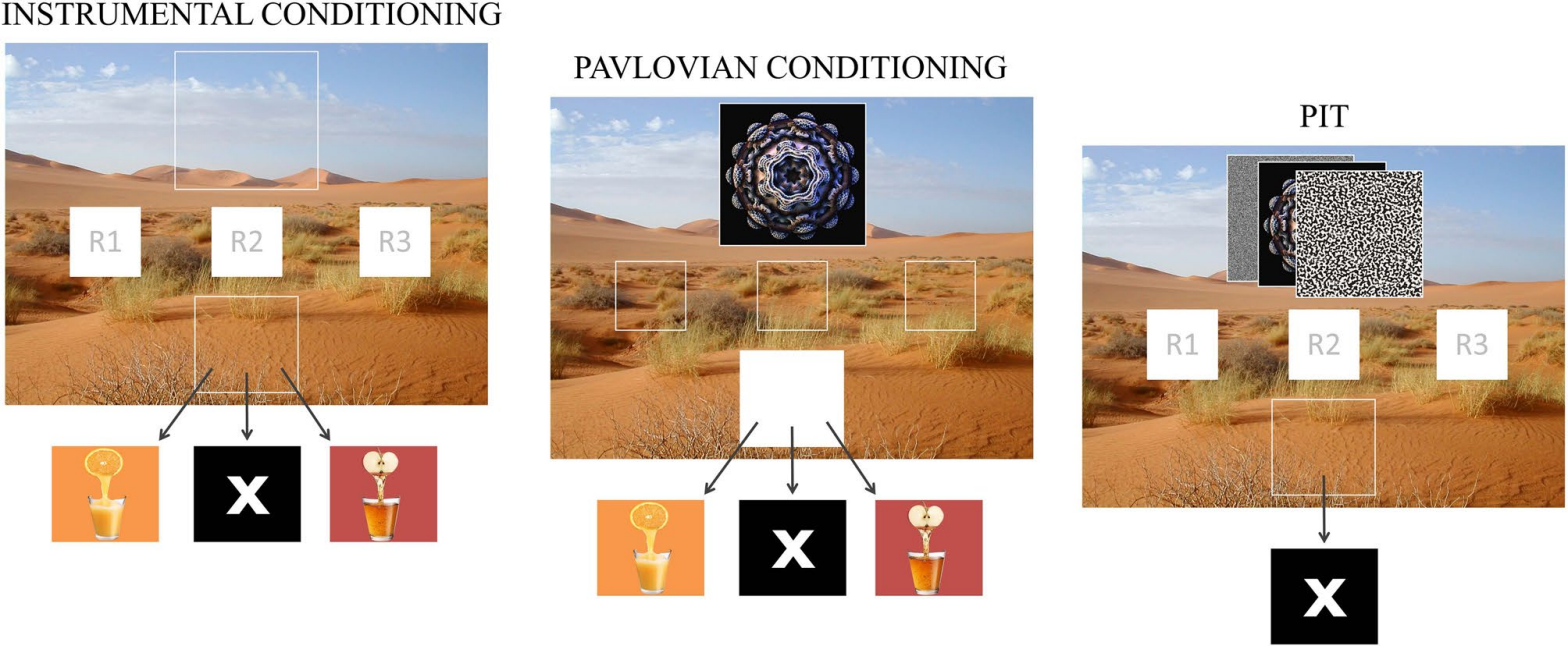
Pavlovian-to-Instrumental Transfer (PIT) task. Visual representation of the three task phases. During instrumental training, participants learned the association between three instrumental responses (R1, R2, R3) and the associated reward (juice) or no-reward (X). Response options were represented by the three middle white squares and outcomes appeared in the bottom square. During Pavlovian training, visual cues (fractals) appeared in the top square and their associated outcomes in the bottom square. During the transfer test, participants performed the instrumental choices under extinction (i.e., in the absence of rewards), while being presented with task-irrelevant Pavlovian cues. As a control condition (mask-only), no CS was presented between the two masking images. In Experiment 1 the cues were presented for either 600 ms (explicit trial) or 16 ms (subliminal trial); in Experiment 2 the cues were presented for either 600 ms (explicit trial) or 33 ms (subliminal trial); in Experiment 3 the cues were presented for 33 ms only. The grey letters (R1, R2, and R3) are for illustrative purposes of the response options and were not actually visible to the participants.

The goal of the present study is to test whether the subliminal processing of a visual cue, previously paired with a reward, is sufficient to favor choices that can lead to the same or a similar reward. To this aim, two experiments compared the effect on choice of subliminal and supraliminal presentation of reward-associated cues, during the transfer phase of a Pavlovian to Instrumental Transfer (PIT) task. The PIT task mirrored the structure used in previous studies^13–18^ and included three phases. First, during instrumental training the participants learned different response-outcome associations. Namely, two instrumental responses were reinforced with two different food rewards (R + 1, R + 2) and one with no food (R−). Second, Pavlovian training follows, during which the participants learned different cue-outcome associations. Namely, two visual cues were paired with the same food rewards previously earned (CS + 1, CS + 2) and one with no food (CS−). Third, during transfer, the influence of task-irrelevant Pavlovian cues on instrumental responding was tested. Instrumental responses were performed under extinction, while the task-irrelevant Pavlovian cues were concurrently presented, allowing to test their influence on choice (Fig. 1, Table 1). Within this scenario, the Pavlovian cues can either bias choice towards the same (i.e. outcome-specific transfer) or a similar (i.e. general transfer) reward. In outcome-specific transfer the cue and the response share a link to the exact same reward (i.e. they were previously connected to physically and sensorially equal rewards), in general transfer the cue and the response only share the appetitive value (i.e. they were previously connected to hedonically similar but sensorially different rewards). For example, the presence of a cue predicting the delivery of dark chocolate can either increase choices to obtain precisely dark chocolate (outcome-specific transfer) or any kind of food (general transfer).

**Table 1:** Trial composition for all task phases.

Task phase	Contingency
Instrumental training	R+_1_ → O1
	R+_2_ → O2
	R− → NR
Pavlovian training	CS+_1_ → O1
	CS+_2_ → O2
	CS− → NR
Outcome-specific transfer	CS+_1_: R_1_ (congruent) versus R_2_ (incongruent)
	CS+_2_: R_1_ (incongruent) versus R_2_ (congruent)
General transfer	CS+_1_: R_2_ versus R− versus CS−: R_2_ versus R−
	CS+_2_: R_1_ versus R− versus CS−: R_1_ versus R−
Mask only	Mask: R_1_ versus R_2_
	Mask: R_1_ versus R−
	Mask: R_2_ versus R−

R, response; CS, conditioned stimulus; O, outcome; +, rewarded; −, unrewarded; NR, no reward.

Experiment 1 tested cue-guided choice when Pavlovian cues were presented for 16 ms (subliminal processing—imperceptible cues) or 600 ms (supraliminal processing). Experiment 2 tested cue-guided choice when Pavlovian cues were presented for 33 ms (subliminal processing—perceptible cues) or 600 ms (supraliminal processing). Finally, in a third control experiment (Experiment 3), Pavlovian cues were presented for 33 ms, and participants reported whether or not they saw the cues, to compare responses to “seen” and “unseen” cues and clarify the role of subjective perception on cue-guided choice.

Evidence from the PIT literature shows behavioral^13–15,19^ and neural^16–18,20–22^ dissociations between outcome-specific and general transfer. While general transfer is more dependent on the motivational properties of the Pavlovian cue, outcome-specific transfer is more related to the sensory-specific properties of the cue^23^. Thus, we expected that the lower level of elaboration consequent to the subliminal presentation of Pavlovian cues^5,11^ may be sufficient to elicit general but not outcome-specific transfer. In other words, we expected the supraliminal processing condition to induce both general and outcome-specific transfer, while the subliminal processing condition to induce general transfer only.

## Results

For each experiment, we first report the analyses on performance during Instrumental and Pavlovian training to confirm that participants correctly learned response-outcome contingencies (Instrumental training) and cue-outcome contingencies (Pavlovian training). Then we report the results on outcome-specific and general transfer. The models used in each analysis are the same across the 3 experiments. Therefore, we report a detailed description of the models only in Experiment 1.

### Experiment 1

#### Instrumental training

##### Number of responses

**Figure 2:**
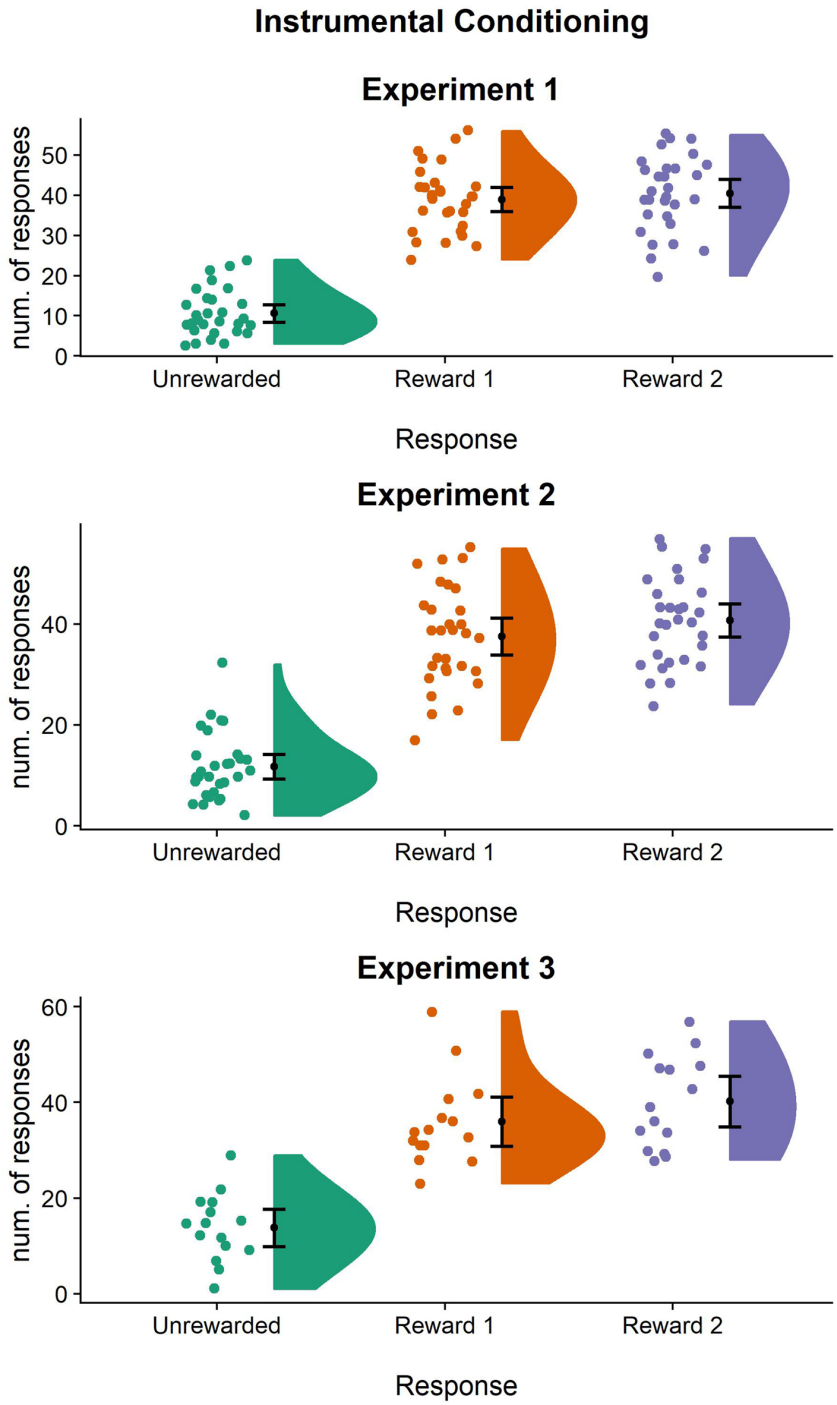
Instrumental training across the three experiments. Number of responses performed during instrumental training in Experiment 1 (top), Experiment 2 (middle), and Experiment 3 (bottom). For each condition, raw individual data are presented on the left and data distribution on the right. The black dots represent the sample mean and the bars represent the 95% confidence interval. Overall, these results confirm that participants correctly learned instrumental contingencies across the three experiments.

To test the learning of instrumental contingencies, the number of rewarded (R+_1_, R+_2_) and unrewarded (R−) responses (mouse clicks) performed during each 10-s trial was compared^16,24^. A linear mixed-effects model was used, with the response (R+_1_/R+_2_/R−) as the independent variable and the number of responses performed as the dependent variable. Results showed a main effect of response (F_(1.58,45.81)_ = 91.61; *p* < 0.0001; part.η^2^ = 0.76; BF_10_ = 2.07e+24). Post-hoc analysis showed a significantly higher number of R+_1_ (mean = 38.93; SD = 1.43) compared to R− (mean = 10.6; SD = 1.43) [β = 28.33; *p* < 0.0001; BF_10_ = 0.31], and a higher number of R+_2_ (mean = 40.46; SD = 1.43) compared to R− [β = 29.87; *p* < 0.0001; BF_10_ = 1.68], with clearly separate 95% confidence intervals (Fig. 2). Critically, no difference between the two rewarded responses emerged [β = 1.53; *p* = 1; BF_10_ = 1.36]. These results indicate a strong preference for both reward-paired responses (R+_1_ and R+_2_) as compared to the unrewarded response (R−), thus showing successful instrumental learning (Fig. 2).

##### Explicit learning

An analysis of explicit associations showed on average 77.01% correct response-outcome associations.

#### Pavlovian training

To test Pavlovian learning, two measures were considered: reaction times to the presentation of the patch preceding the outcome and the pre-post change in the liking of the conditioned cues (CSs).

##### Reaction times

A Kruskal–Wallis rank-sum test with CS (CS+_1_/CS+_2_/CS−) as the independent variable and reaction times (in milliseconds) as the dependent variable revealed a significant main effect of CS (X^2^_(2)_ = 7.61; *p* = 0.02; BF_10_ = 1.16). Post-hoc comparisons showed that reaction times were significantly faster for CS+_1_ (mean = 590.14; SD = 749.99) compared to CS− (mean = 670.450; SD = 837.52) [*p* = 0.006; BF_10_ = 1.22], without significant difference between CS+_2_ (mean = 558.24; SD = 752.30) and CS− [*p* = 0.26; BF_10_ = 0.29]. Crucially, there was no difference between CS + 1 and CS + 2 [*p* = 0.09; BF_10_ = 0.08]. Although confidence intervals slightly overlapped, participants showed a tendency to be faster when a reward was anticipated (CS + 1, CS + 2) as compared to when no reward was expected (CS−), indicating discrimination between the cues and, thus, Pavlovian training.

##### CS liking

**Figure 3:**
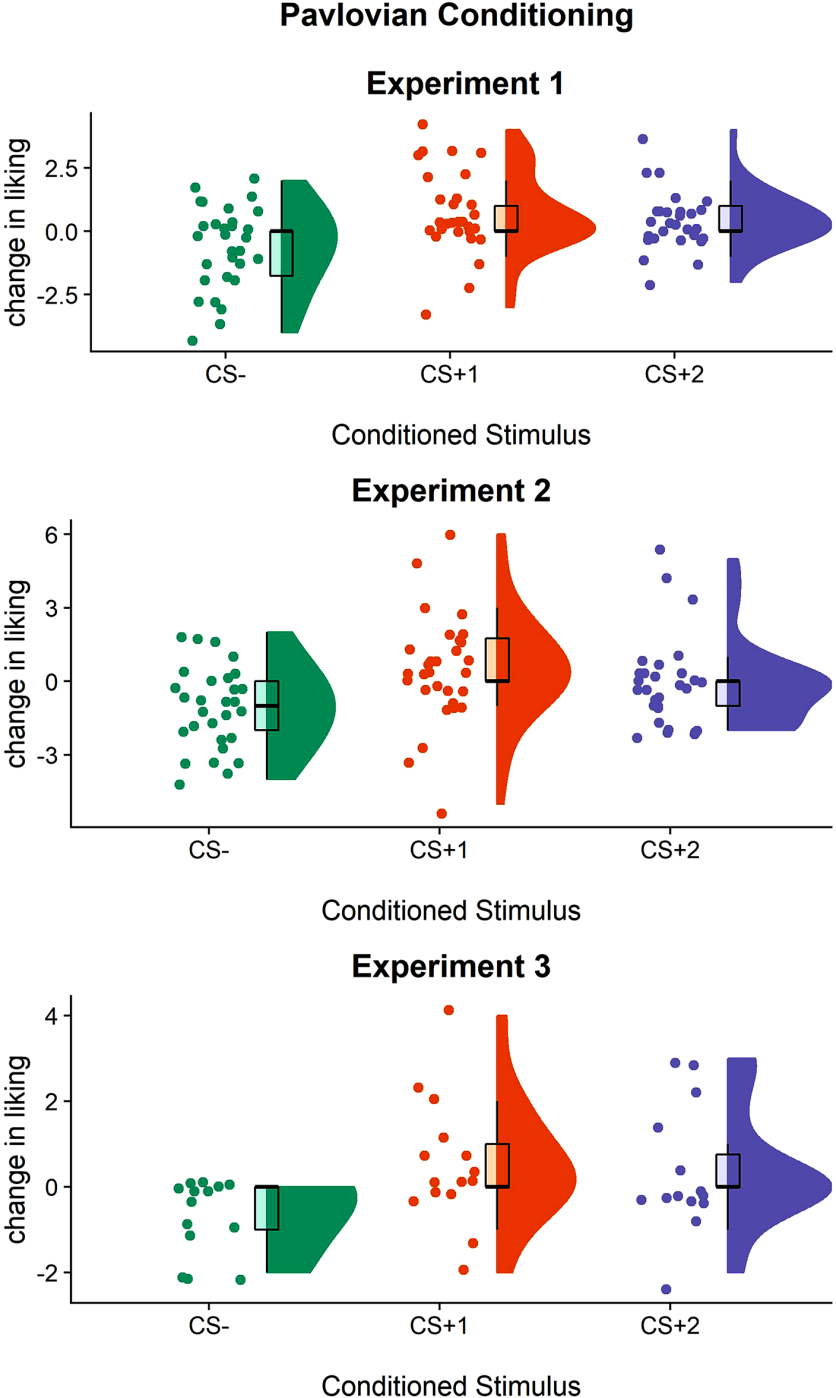
Pavlovian training across the three experiments. Change in liking calculated as liking of the visual cue (post–pre) Pavlovian training in Experiment 1 (top), Experiment 2 (middle), and Experiment 3 (bottom). For each condition, raw individual data are presented on the left and data distribution on the right. Boxplots with median and interquartile range are depicted in black. Overall, these results confirm that participants correctly learned Pavlovian contingencies across the three experiments.

A Kruskal–Wallis rank-sum test with CS (CS+_1_/CS+_2_/CS−) as the independent variable and change in CS liking (rating post–rating pre) as the dependent variable revealed a significant main effect of CS (X^2^_(2)_ = 21.151; *p* < 0.001; BF_10_ = 12,657.55; e = 0.02%). Post-hoc comparisons showed significant increase in liking for CS+_1_ (mean = 0.65; SD = 1.52) compared to CS− (mean = − 0.66; SD = 1.61) [*p* < 0.0001; BF_10_ = 1,422.607], and in liking for CS+_2_ (mean = 0.44; SD = 1.07) compared to CS− [*p* < 0.0001; BF_10_ = 775.60], with clearly separate 95% confidence intervals (Fig. 3). Crucially, there was no difference between CS + 1 and CS + 2 [*p* = 0.57; BF_10_ = 0.27]. These results indicated that, after Pavlovian training, participants significantly increased their liking for reward-paired cues (CS+_1_ and CS+_2_), as compared to the unrewarded cue (CS−), thus confirming successful Pavlovian training (Fig. 3).

##### Explicit learning

An analysis of explicit associations showed on average 88.5% correct cue-outcome associations.

#### Transfer effect

##### Outcome-specific transfer

**Figure 4:**
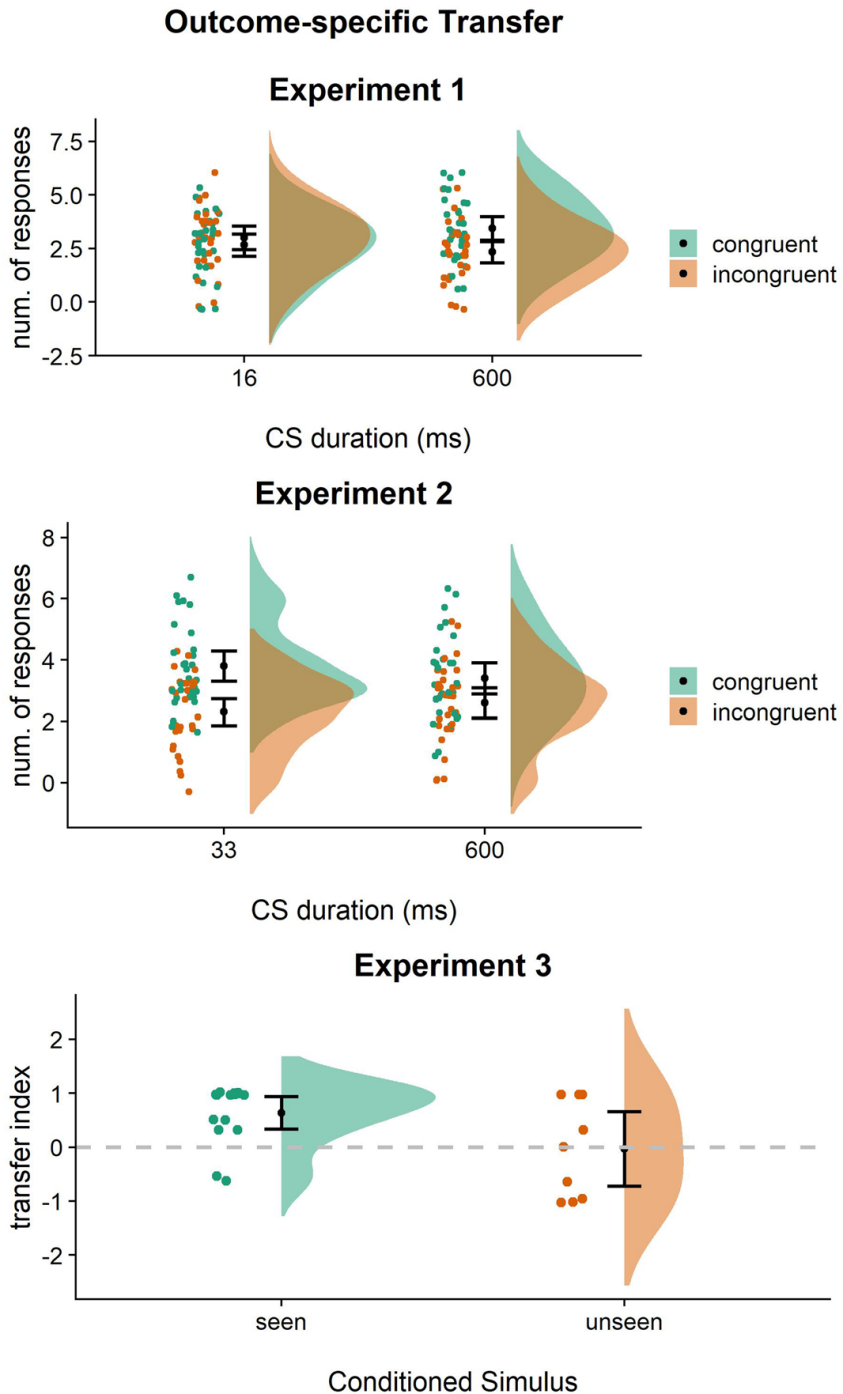
Outcome-specific transfer across the three experiments. Number of congruent and incongruent responses performed during the transfer phase of Experiment 1 (top) and Experiment 2 (middle) and transfer index reported in Experiment 3 (bottom). In the transfer index, values > 0 indicate a prevalence of congruent over incongruent responses and vice versa. For each condition, raw individual data are presented on the left and data distribution on the right. The black dots represent the sample mean and the bars represent the 95% confidence interval. Overall, these results show that the supraliminal (600 ms) presentation of Pavlovian cues produced outcome-specific transfer, while the subliminal presentation did not (16 ms and 33 ms-seen).

A linear mixed-effects model was used with cue duration (16 ms/600 ms) and kind of response (congruent/incongruent) as the independent variables, and the number of responses as the dependent variable. Results showed statistically significant duration by response interaction (F_(1,29)_ = 5.00; *p* = 0.03; part. η^2^ = 0.15; BF_10_ = 6,117,174). All other effects were not statistically significant (*p* > 0.21). Post-hoc analysis showed a significantly higher number of congruent (mean = 3.43; SD = 1.47) than incongruent (mean = 2.33; SD = 1.34) responses when the CS was presented for 600 ms [β = 1.1; *p* < 0.001; BF_10_ = 10.16], but not when it was presented for 16 ms (*p* = 1; BF_10_ = 0.369) [β = 0.33; *p* = 1; BF_10_ = 0.36]. Although confidence intervals slightly overlap (Fig. 4), participants showed a significant increase of congruent over incongruent responses in the 600 ms condition only. Thus, outcome-specific transfer was observed only when the reward-associated Pavlovian cue was presented supraliminally (600 ms), but not when it was presented subliminally (16 ms).

##### General transfer

**Figure 5:**
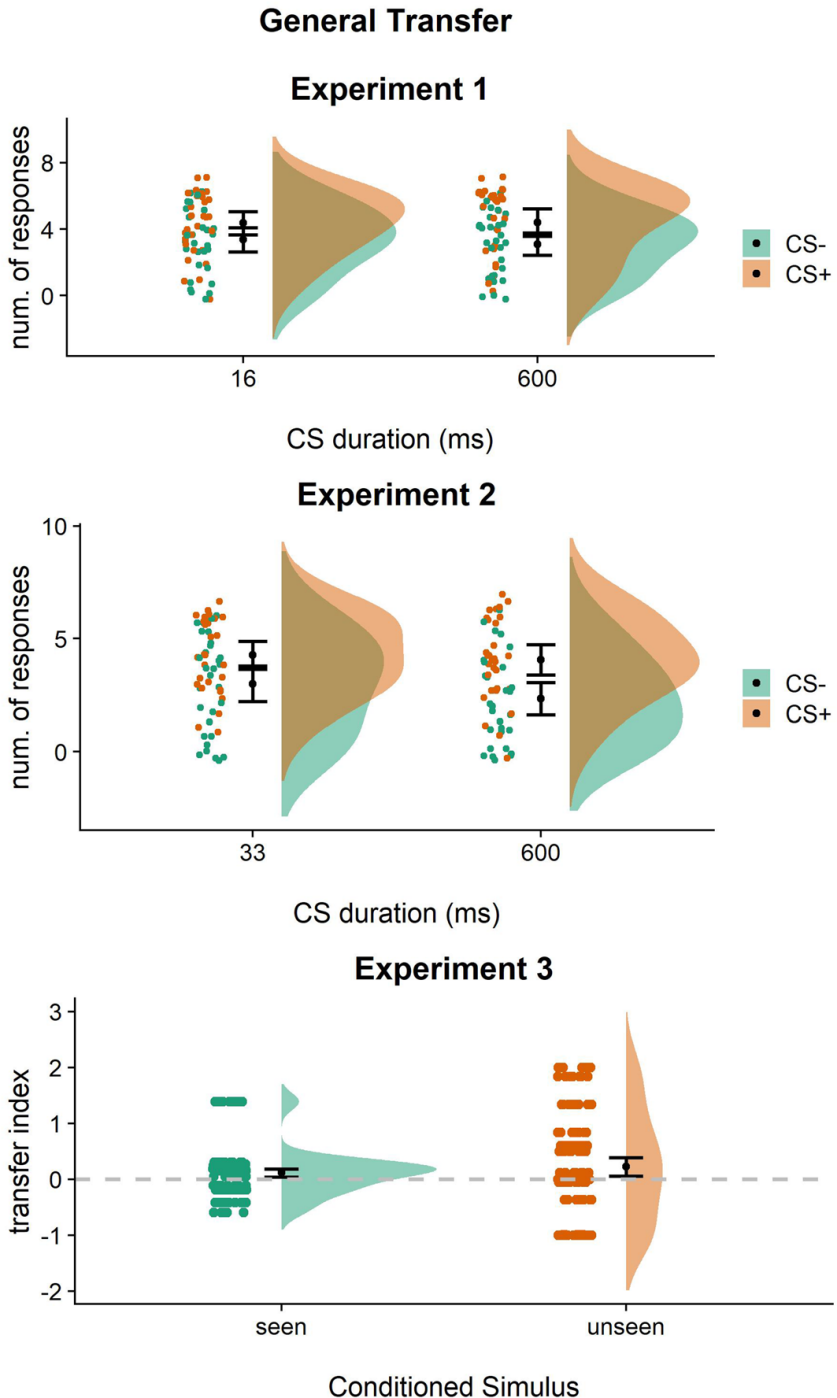
Outcome-specific transfer across the three experiments. Number of responses performed during the presentation of reward-associated (CS+) and unrewarded (CS−) cue during the transfer phase of Experiment 1 (top) and Experiment 2 (middle) and transfer index reported in Experiment 3 (bottom). In the transfer index, values > 0 indicate a prevalence of rewarded over unrewarded responses during CS+ trials and vice versa. For each condition, raw individual data are presented on the left and data distribution on the right. The black dots represent the sample mean and the bars represent the 95% confidence interval. Overall, these results show that both the supraliminal (600 ms) and subliminal (16 ms and 33 ms) presentation of Pavlovian cues produced general transfer.

A linear mixed-effects model was used with cue duration (16 ms/600 ms) and CS (CS+/CS−) as the independent variables, and the number of rewarded responses as the dependent variable. Results showed a statistically significant main effect of CS (F_(1,29)_ = 11.22; *p* = 0.002; part.η^2^ = 0.28; BF_10_ = 1.135695; err% = 1.28). All other effects were not statistically significant (*p* > 0.43). Although confidence intervals slightly overlapped (Fig. 5), participants performed a higher number of rewarded responses when presented with the CS+ (mean = 4.38; SD = 2.01) than the CS− (mean = 3.23; SD = 1.86), irrespective of the cue duration [β = 1.15; *p* = 0.002; BF_10_ = 19.88]. Thus, general transfer was observed both when the reward-associated Pavlovian cue was presented supraliminally (600 ms) and when it was subliminally presented (16 ms).

Mask-only. As a further control condition, the number of responses performed during the transfer phase was compared between mask-only trials (in which no Pavlovian cue was presented between the two masking images) and those in which a Pavlovian cue was presented for a subliminal (16 ms) or supraliminal (600 ms) duration. A linear mixed-effects model, with the cue (none/16 ms/600 ms) as the independent variable and number of responses as the dependent variable, showed a significant effect (F_(1,36,39.32)_ = 192.25; *p* < 0.0001; part.η^2^ = 0.87; BF_10_ = 52,723,772). Post-hoc analysis revealed a significantly (*p* < 0.0001) lower number of responses during trials in which no Pavlovian cue was presented (mean = 10.72; SD = 5.81) than in trials where the Pavlovian cue was presented for either 16 ms (mean = 16.78; SD = 7.31) or 600 ms (mean = 17.00; SD = 7.29). This result confirmed an increase in responding when the participant was presented with a Pavlovian cue.

### Experiment 2

#### Instrumental training

##### Number of responses

Results showed a main effect of response (F_(1.68,48.80)_ = 69.88; *p* < 0.0001; part.η^2^ = 0.71; BF_10_ = 4.46 + 20). Post-hoc analysis revealed a significantly higher number of R+_1_ (mean = 37.53; SD = 9.86) compared to R− (mean = 11.73; SD = 6.58) [β = 25.8; *p* < 0.0001; BF_10_ = 0.54], and of R+_2_ (mean = 40.73; SD = 8.73) compared to R− [β = 29; *p* < 0.0001; BF_10_ = 1.07], with clearly separate 95% confidence intervals. Critically, no difference between the two rewarded responses emerged [β = 3.2; *p* = 0.71; BF_10_ = 4.54]. These results indicate a preference for both reward-paired responses (R+_1_ and R+_2_) as compared to the unrewarded response (R−), thus indicating successful instrumental learning (Fig. 2).

##### Explicit learning

An analysis of explicit associations showed on average 77.33% correct response-outcome associations.

#### Pavlovian training

##### Reaction times

Results showed a significant main effect of CS (X^2^_(2)_ = 11.81; *p* = 0.002; BF_10_ = 1.13; e = 0.01%). Post-hoc comparisons revealed that reaction times were significantly faster for CS+_1_ (mean = 558.21; SD = 631.54) compared to CS− (mean = 668.62; SD = 735.22) [*p* = 0.004; BF_10_ = 2.99], and for CS+_2_ (mean = 562.01; SD = 635.56) compared to CS− [*p* = 0.002; BF_10_ = 2.26]. Crucially, there was no difference between CS + 1 and CS + 2 [*p* = 0.82; BF_10_ = 0.06]. Although confidence intervals slightly overlapped, overall participants showed a tendency to be faster when a reward was anticipated (CS + 1, CS + 2) as compared to when no reward was expected (CS−), thus indicating discrimination between the cues and, thus, Pavlovian training.

##### CS liking

A Kruskal–Wallis rank-sum test was used, with CS (CS+_1_/CS+_2_/CS−) as the independent variable and change in CS liking (rating post–rating pre) as the dependent variable. Results showed a significant main effect of CS (X^2^_(2)_ = 22.32; *p* < 0.001; BF_10_ = 399.69). Post-hoc comparisons showed a significant increase in liking for CS+_1_ (mean = 0.53; SD = 2.17) compared to CS− (mean = − 1.03; SD = 1.64) [*p* < 0.01; BF_10_ = 797.15], and for CS+_2_ (mean = − 1.03; SD = 1.64) compared to CS− [*p* = 0.01; BF_10_ = 7.28], with clearly separate 95% confidence intervals (Fig. 3), Crucially, there was no difference between CS + 1 and CS+2 [*p* = 0.007; BF_10_ = 0.27]. These results indicated that, after Pavlovian training, participants significantly increased their liking for reward-paired cues (CS+_1_ and CS+_2_), as compared to the unrewarded cue (CS−), thus confirming successful Pavlovian training (Fig. 3).

##### Explicit learning

An analysis of explicit associations showed on average 84.52% correct cue-outcome associations.

#### Transfer effect

Outcome-specific transfer. Results showed a statistically significant main effect of response (F_(1,29)_ = 8.01; β = 1.15; *p* = 0.008; part.η^2^ = 0.22; BF_10_ = 3,738.136). All other effects were not statistically significant (*p* > 0.14). Although confidence intervals slightly overlap (Fig. 4), participants performed a higher number of congruent responses (mean = 3.60; SD = 1.35) than incongruent responses (mean = 2.45; SD = 1.25) irrespective of the cue duration. Thus, outcome-specific transfer was observed both when the reward-associated Pavlovian cue was presented supraliminally (600 ms) and when it was presented subliminally but perceptible (33 ms).

##### General transfer

A statistically significant main effect of the kind of cue was found (F_(1,29)_ = 21.51; β = 1.5; *p* = 0.0001; part.η^2^ = 0.43; BF_10_ = 697.184). All other effects were not statistically significant (*p* > 0.09). Although confidence intervals slightly overlap (Fig. 5), participants performed a higher number of rewarded responses when presented with the CS+ (mean = 4.16; SD = 1.72) than the CS− (mean = 2.66; SD = 2.02) irrespective of the cue duration [β = 1.5; *p* = 0.0001; BF_10_ = 697.18]. Thus, general transfer was observed both when the reward-associated Pavlovian cue was presented supraliminally (600 ms) and when it was presented subliminally but perceptible (33 ms).

##### Mask-only

Results showed a significant effect (F_(1.36,39.32)_ = 387.10; *p* < 0.0001; part.η^2^ = 0.93; BF_10_ = 20,640,516,176). Post-hoc analysis revealed a significantly (*p* < 0.0001) lower number of responses when no Pavlovian cue was presented (mean = 10.38; SD = 5.31) compared to trials in which the Pavlovian cue was presented for either 33 ms (mean = 16.98; SD = 7.00) or 600 ms (mean = 17.18; SD = 7.01). This result confirmed a selective increase in responding when the participants were presented with a Pavlovian cue.

#### Interim discussion

Overall, the results of experiments 1 and 2 show that supraliminal (600 ms) reward-associated Pavlovian cues biased choice towards motivationally similar outcomes (i.e., general transfer) as well as outcomes with the same sensory-specific properties (i.e., outcome-specific transfer), in line with previous evidence^8,14,15,24^. The novel result is that the subliminal (16 ms and 33 ms) presentation of reward-associated Pavlovian cues biased choice towards motivationally similar outcomes (i.e., general transfer). Additionally, while Experiment 1 showed no outcome-specific transfer for the 16 ms condition (subliminal processing—imperceptible cues), Experiment 2 reported such effect for cues lasting 33 ms (subliminal processing—perceptible cues). Thus, the extent to which subliminally presented reward-associated Pavlovian cues influence choice towards outcomes with the same sensory-specific properties remains to be clarified. These results might be explained by the individual variability of the subjective visibility with a subliminal presentation of 33 ms. Indeed, studies comparing behavioral responses and brain activation indicated that a proportion of cues presented for 33 ms could still be categorized as clearly visible^11^. In this case, a precise differentiation between seen and unseen trials could help clarifying the results from Experiment 2. For this reason, in a third experiment, the PIT paradigm was implemented using only the subliminal presentation of 33 ms, and the transfer effect was compared between “seen” and “unseen” trials, as assessed by participants’ judgment of their subjective experience (see “Methods”).

### Experiment 3

#### Instrumental training

##### Number of responses

Results showed a main effect of response (F_(1.79,25.06)_ = 26.64; *p* < 0.0001; part.η^2^ = 0.66; BF_10_ = 41,190,683). Post-hoc analysis showed a significantly higher number of R+_1_ (mean = 36; SD = 9.24) compared to R− (mean = 13.8; SD = 7.06) [β = 22.2; *p* < 0.0001; BF_10_ = 0.50], and a of R+_2_ (mean = 40.02; SD = 9.55) compared to R− [β = 26.3; *p* < 0.0001; BF_10_ = 187,735.6]. Critically, no preference between the two rewarded responses emerged [β = 4.2; *p* = 0.86; BF_10_ = 2,969,415]. These results indicate a preference for both reward-paired responses (R+_1_ and R+_2_) as compared to the unrewarded response (R−), thus indicating successful instrumental learning (Fig. 2).

##### Explicit learning

Analysis of explicit associations showed on average 73.8% correct response-outcome associations.

#### Pavlovian training

##### Reaction times

Results showed a significant main effect of CS (X^2^_(2)_ = 6.06; *p* = 0.04; BF_10_ = 37.49). Post-hoc comparisons showed significantly faster reaction times for CS+_1_ (mean = 344.56; SD = 227.21) compared to CS− (mean = 451.37; SD = 443.36) [*p* = 0.01; BF_10_ = 71.53], and faster reaction times for CS+_2_ (mean = 379.86; SD = 268.74) compared to CS− [*p* = 0.4; BF_10_ = 1.46]. Crucially, there was no difference between CS + 1 and CS + 2 [*p* = 0.12; BF_10_ = 0.39]. Participants showed a tendency to be faster when a reward was anticipated (CS + 1, CS + 2) as compared to when no reward was expected (CS−), indicating discrimination between the cues and, thus, Pavlovian training.

##### CS liking

Results showed a significant main effect of CS (X^2^_(2)_ = 16.073; *p* < 0.001; BF_10_ = 46.66; err% = 0.01). Post-hoc comparisons revealed a significant (*p* < 0.001) increase in liking for CS+_1_ (mean = 0.53; SD = 1.38) compared to CS− (mean = − 0.64; SD = 0.82), and in liking for CS+_2_ (mean = 0.42; SD = 1.37) compared to CS−, but no difference between CS + 1 and CS + 2 (*p* = 0.61). These results indicated that, after Pavlovian training, participants significantly increased their liking for reward-paired cues (CS+_1_ and CS+_2_), as compared to the no-reward cue (CS−), thus confirming effective Pavlovian training.

A Kruskal–Wallis rank-sum test with CS (CS+_1_/CS+_2_/CS−) as the independent variable and change in CS liking (rating post–rating pre) as the dependent variable showed a significant main effect of CS (X^2^_(2)_ = 16.073; *p* < 0.001; BF_10_ = 46.66; err% = 0.01). Post-hoc comparisons showed an increase in liking for CS+_1_ (mean = 0.53; SD = 1.38) compared to CS− (mean = − 0.64; SD = 0.82) [*p* < 0.01; BF_10_ = 96.21], and an increase in liking for CS+_2_ (mean = 0.42; SD = 1.37) compared to CS− [*p* = 0.001; BF_10_ = 36.11], with clearly separate 95% confidence intervals (Fig. 3), Crucially, there was no difference between CS + 1 and CS + 2 [*p* = 0.61; BF_10_ = 0.27]. These results indicated that, after Pavlovian training, participants significantly increased their liking for reward-paired cues (CS+_1_ and CS+_2_), as compared to the unrewarded cue (CS−), thus confirming successful Pavlovian training (Fig. 3).

##### Explicit learning

Analysis of explicit associations showed on average 68% correct cue-outcome associations.

#### Transfer effect

On a total of 72 trials, 71% were categorized as “seen” (average number = 51) and 34% were categorized as “unseen” (average number = 25).

Outcome-specific transfer. To confirm the presence of the outcome-specific transfer, the transfer index was tested against zero, where zero represented an equal number of congruent and incongruent responses, i.e. absence of transfer. A higher transfer index (indicating a higher number of congruent than incongruent responses) was reported during seen trials (t_(14)_ = 4.49; *p* = 0.001; 95% CI [0.33, 0.94]; BF_10_ = 69.67) but not during unseen trials (t_(14)_ = − 0.09; *p* = 0.9; 95% CI [− 0.72, 0.66]; BF_10_ = 0.32) was reported (Fig. 4). In a second analysis, a linear mixed-effects model was used with CS (seen/unseen) as the independent variable and the outcome-specific transfer index as the dependent variable. Results showed a statistically significant difference (F_(1,14)_ = 7.13; β = 0.84; *p* = 0.03; part.η^2^ = 0.47; BF_10_ = 2.18) with a higher transfer index during seen (mean = 0.63; SD = 0.54) than unseen (mean = − 0.03; SD = 0.89) trials (Fig. 4). Thus, outcome-specific transfer was observed only when the reward-associated Pavlovian cue was explicitly perceived (seen), but not when it was not explicitly perceived (unseen). Overall, these results demonstrate that outcome-specific transfer depends on the conscious elaboration of the reward-associated cue.

##### General transfer

To confirm the presence of general transfer, all values were tested against zero, where zero represented the absence of general transfer. During both seen (t_(14)_ = 3.2; *p* = 0.003; 95% CI [0.04, 0.18]; BF_10_ = 12.03) and unseen (t_(14)_ =  − 2.67; *p* = 0.008; 95% CI [0.05, 0.38]; BF_10_ = 2.90) trials, the number of responses was higher during CS+ than during CS− (seen mean = 0.11, SD = 0.41; unseen mean = 0.22, SD = 0.97) (Fig. 5). In a second analysis, a linear mixed-effects model was used with CS (seen/unseen) as the independent variable and the general transfer index as the dependent variable. Results showed no significant difference (F_(1,14)_ = 0.34; β = 0.19; *p* = 0.57; part.η^2^ = 0.03; BF_10_ = 0.28) between seen (mean = 0.11; SD = 0.41) and unseen (mean = 0.22; SD = 0.97) trials (Fig. 5). Thus, general transfer was observed both when the reward-associated Pavlovian cue was explicitly perceived (seen) and when it was not explicitly perceived (unseen). Overall, these results demonstrate that general transfer independent of the conscious elaboration of the reward-associated cue.

## Discussion

The present work investigated the extent to which conscious elaboration represents a boundary condition within which environmental cues can influence individual choice. To this end, we compared the effect of subliminal and supraliminal presentation of reward-associated Pavlovian cues during the transfer phase of a Pavlovian-to-Instrumental Transfer (PIT) task. In line with previous evidence^2,8,14,15, 24,25^, the supraliminal presentation (600 ms) of reward-associated cues biased choice towards rewards sharing the precise sensory-specific (outcome-specific transfer) and motivationally similar (general transfer) properties of the cues. On the other hand, the subliminal presentation of reward-associated cues was always able to bias choice towards motivationally similar rewards (general transfer), but not sufficient to bias choices associated with rewards sharing the precise sensory-specific properties of the cue (outcome-specific transfer). More precisely, perceptible subliminal Pavlovian cues (33 ms) triggered outcome-specific transfer only when the cues were explicitly perceived (seen trials), but not when they were only subliminally processed (unseen trials). Crucially, imperceptible subliminal Pavlovian cues (16 ms) were never associated with outcome-specific transfer.

Taken together, the present findings suggest that cue-guided choices are modulated by the level of perceptual threshold (i.e., subliminal vs supraliminal) of reward-associated cues^14,15,19^. While a conscious elaboration of the reward-associated cue is necessary to bias choice towards the exact same reward, subliminal processing is only sufficient to bias choice towards rewards sharing the same motivational properties.

The present results provide crucial evidence for the theoretical accounts of cue-guided choice. This study supports previous evidence highlighting behavioral dissociations at the core of outcome-specific and general transfer^14,15,19^. For example, high-level cognitive abilities, such as working memory, appear to be crucial for the expression of a sensory specific biasing effect, but not of a general biasing effect^14^. In line with this, human neuroimaging studies reported the activation of separate brain regions for outcome-specific (dorsal striatum and ventral amygdala) and general (ventral striatum and dorsal amygdala) transfer^16–18,20,22^. Such differences could be attributed either to separate mechanisms, possibly mediated by different neural networks, or to a continuum of the same system ending on a different path as a function of quantity and quality of the information carried by the cue. While a lower level of processing provided by the subliminal presentation of the cue^5,11^ might be sufficient to convey the necessary motivational properties responsible for general transfer, a higher level of processing would be required to activate the sensory-specific representation of the reward characterizing outcome-specific transfer. At least in humans, outcome-specific and general transfer could thus be supported by different mechanisms, one more explicit and another more automatic^14,15,19,23^. Future studies might explore whether these differences can be related to the activation of different brain networks. For example, the fronto-striatal loops involved in flexible behavioral adaptation^21,26–30^ could be differentiated in a cortical component related to supraliminal processing, and a subcortical component involved in subliminal processing^16,23,25,31,32^. Another critical issue is whether individual differences in the prevalence of the automatic component over the explicit one can indicate a higher predisposition to develop a maladaptive behavior^13,33^.

These results have implications in both daily life choices and clinical contexts. In everyday life, this result translates to the fact that although an explicitly perceived logo can persuade to buy a specific product (e.g., viewing the McDonald’s logo along the street can lead to driving the car towards a McDonald fast-food restaurant), a much lower level of processing of the same information can still bias choice towards the purchase of similar products (e.g., the presence of McDonald’s logo on the street, even if not consciously perceived, can lead to driving the car towards any fast-food restaurant). From a clinical perspective, cue-guided choices are related to several conditions, ranging from compulsive conducts, to neuropsychiatric disorders (like depression), and addiction (for its strong implications with relapse)^7,9,34–43^. The prevalence of automatic responding is indeed generally associated with addictive, impulsive, and obsessive traits^34,39,44–47^. In this sense, the lack of conscious processing that characterizes general transfer could potentially be a marker of vulnerability to maladaptive forms of cue-guided choices^3^. In the case of addiction, for example, although no direct correlation between drug or food forms of addiction and outcome-specific transfer has been reported in literature^48,49^, it cannot be excluded that this is instead related to forms of cue-driven choice requiring a lower level of processing, like in the case of general transfer^50^.

Finally, some limitations of the present study shall be discussed. Differently from previous studies, the participants did not receive an actual reward for themselves (but rather played to quench the thirst of a camel crossing the desert) and were exposed to Pavlovian cues for a short time^13–15^. Although these manipulations may have weakened their motivation to perform the task and could partially explain the presence of small effect sizes, both outcome-specific and general transfer were still observed. Moreover, the operationalization of general transfer in the present study differs from the one adopted in animal literature^25^, thus limiting the translational potential of the results linked to general transfer. However, the same operationalization has been used in the human literature^13–15,17,18^, thus enabling a comparison of our results with previous studies.

## Conclusions

The present findings highlight how environmental cues can influence human choice depending on their level of processing. If explicitly perceived, reward-associated cues can guide behavior towards choices aimed at the same or similar rewards. Crucially, subliminal processing of such cues is sufficient to bias choice towards motivationally similar, yet not identical, rewards. The current understanding of cue-guided choices, and of their implications in daily life and clinical conditions, warrants further studies to explore the extent to which reward-associated cues can exert a maladaptive control over choice. We suggest that the dissociation outlined in this as well as in previous studies^14–20,23,25^ between outcome-specific and general transfer is crucial to shed new light on the mechanisms at the core of cue-guided choices.

## Methods

### Participants

The three experiments were performed on independent samples: Experiment 1 involved 15 females and 15 men, mean ± sd 25.28 ± 2.69; Experiment 2 involved 15 females and 15 men, mean ± sd 24.73 ± 2.58; Experiment 3 involved 8 females and 7 men, mean ± sd 25.73 ± 2.73. All participants gave their written informed consent to take part in the experiment. The study was conducted following institutional guidelines and the 1964 Declaration of Helsinki and was approved by the Ethics Committee of the Department of Psychology of the University of Campania. Participants were naive about the hypotheses of the experiment. All volunteers had no history of neurological or psychiatric diseases were recruited from the student population of the University of Campania “L. Vanvitelli”. The number of participants was determined using power analysis for F test family (power = 0.9, alpha = 0.05, effect size f = 0.35), estimating the effect size based on previous literature using a similar PIT task^13–15^. This analysis was computed using the software G*Power version 3.1.9.4.

### Pavlovian-to-Instrumental Transfer task

The task mirrored the structure used in previous studies^13–18^: (1) Instrumental training, in which participants learned a response-contingent reward; (2) Pavlovian training, in which participants learned a cue-contingent reward; (3) Transfer test, during which the influence of task-irrelevant Pavlovian cues on instrumental responding was tested. The same visual settings were used in all phases of the PIT task (see Fig. 1). A computer running OpenSesame software^51^ controlled cue presentation. Five white (2 mm thickness) squared frames were displayed on a 17-inch color monitor with the image of a desert as background. One frame was positioned in the upper portion of the screen, highlighting the area where the Pavlovian conditioned cues would be displayed during the Pavlovian training and transfer test. Three squares were positioned horizontally next to each other in the middle portion of the screen, highlighting the regions of the screen to be clicked with the mouse to make a response during instrumental training and transfer test. One square was positioned in the bottom portion of the screen, highlighting the area where the outcome would be displayed during instrumental training, Pavlovian training and transfer test.

#### Instrumental training

This training aimed to learn the association between a specific instrumental response and its corresponding outcome. In each trial, participants made a response, i.e. they clicked on one of the three frames placed in the middle portion of the screen. Two responses (R+_1_ and R+_2_) were each paired with one of two different reward outcomes (i.e. the picture of an apple and orange juice) on 80% of trials, and with the no-reward outcome (i.e. picture of an ‘X’) in the remaining 20% of trials. The third response (R−) was always paired with the no-reward outcome. The response-outcome association was counterbalanced across participants. In each trial, only two out of the three responses were available, and this was indicated to participants by a white patch appearing within the square. After each response, a corresponding no-reward or reward outcome appeared for 1 s in the bottom square. The task consisted of a total of 90 randomized trials (30 trials for each pair of responses, see Table 1), and lasted about 5 min. Three example trials (one for each pair of responses) were performed before beginning.

#### Pavlovian training

This training aimed to learn the association between a specific conditioned cue and its corresponding outcome. In each trial, one of three conditioned cues (CS, i.e. fractal images balanced for luminance, complexity, and color saturation) appeared within the upper frame for 2.5 s. Then, a white patch appeared within the bottom square. Participants were instructed to press the left-Ctrl button on the computer keyboard as quickly as possible to remove the patch and discover the outcome hidden behind it. The outcome was presented for 1 s. Two CSs (CS+_1_ and CS+_2_) were associated with a reward (apple or orange juice) on 80% of trials and with the no-reward outcome (i.e. picture of an ‘X’) in the remaining 20% of trials. The third CS (CS−) was associated with the no-reward outcome on all trials. The cue-outcome association was counterbalanced across participants. The task consisted of 60 randomized trials (20 per CS, see Table 1), for a total duration of about 6 min. Three example trials (one for each CS) were performed before beginning.

This Pavlovian speeded reaction-time response has been successfully used to obtain a behavioral measure of Pavlovian training^13,14,16^. To avoid a possible instrumental component, participants were explicitly told and demonstrated that the reward delivery was not dependent upon their response. Indeed, even without a button press, the patch would disappear after 3 s and reveal the hidden outcome. The main reason for using a speeded response was to mirror PIT studies on animals, in which Pavlovian training is measured by a behavior performed to gain the reward^31,52,53^. The rationale is that the higher arousal produced by reward expectancy shall produce faster reaction times when a reward rather than no reward is predicted (i.e. CS+ vs. CS−)^13,14, 16,31,52,53^. As a further measure of learning, subjective liking for each CSs was rated by participants on a 9-items Likert scale, before and after Pavlovian training. The rationale was to observe an increase in liking scores after Pavlovian training for CS+_1_ and CS+_2_, as compared to the CS−. At the end of the two training sessions, explicit learning was assessed. Participants saw each response option and CS and were required to associate them with their corresponding outcomes.

#### Transfer test

This phase aimed to test the influence of the Pavlovian CSs to drive instrumental responses. The task mirrored instrumental training, with two differences. First, while performing responses, task-irrelevant Pavlovian CSs were presented within the upper square, at the beginning of the trial. Crucially, the duration (supraliminal/subliminal) of the CSs varied across the three experiments: in Experiment 1, the cues lasted for either 600 ms (supraliminal processing) or 16 ms (subliminal processing–imperceptible cues); in Experiment 2, the cues lasted for either 600 ms (supraliminal processing) or 33 ms (subliminal processing–perceptible cues); in Experiment 3, all cues lasted for 33 ms. In a control condition (mask-only), no CS was presented between the two masking images. Only in Experiment 3, after each response participants were presented with all CSs and a “no-image” option and invited to indicate whether one of the images appeared in the previous trial. Trials of the PIT phase were consequently categorized as “seen” if the CS was correctly recognized, or “unseen” if the CS was either not correctly recognized or the no-image option was selected. Second, the whole task was performed under extinction, so that no reward was ever delivered. Extinction is a standard procedure for assessing transfer effect, both in human and non-human animal research, since it allows to test the influence of Pavlovian cues on instrumental responding without the confounding effects of the reward^16,18,54^.

### Subliminal presentation of visual cues

Experimentally, the conscious perception of a target visual cue occurs only if the interval between two masking images preceding and following the target exceeds a certain duration^5,11,55^. The threshold of such duration has been widely investigated and is commonly held to range between 16 and 33ms^5, 11,55^. At a duration of 16 ms, visual cues are not accessible to consciousness and are imperceptible; at a duration of 33 ms they are still not accessible to consciousness but perceptible^5,11^. A duration of 600 ms allows supraliminal processing^5,11,55^. In this experiment, a backward masking paradigm with two different masks (random black and white dots) presented immediately before and after the presentation of the cues was used^11,55^ following a pilot study aimed to test the implementation and validity of the chosen duration of the cues (16 ms/33 ms/600 ms). The duration of the masks was equally divided between the two (pre/post cue) and varied according to the duration of the CS so that the total duration of the three cues was always equal to 1 s. For instance, when the target cue lasted 600 ms, the two masks lasted 200 ms each.

### Definition of outcome-specific and general transfer and transfer indexes

#### Outcome-specific transfer

Participants were presented with a task-irrelevant CS+ (CS+_1_ or CS+_2_) while required to choose between two available response options (R+_1_ and R+_2_) previously paired with two different reward outcomes, of which only one was also previously paired with the concurrently presented CS. So, for example, if the CS+_1_ was presented, choosing R+_1_ would constitute a congruent response, while choosing R+_2_ would constitute an incongruent response. Similarly, if the CS+_2_ was presented, choosing R+_1_ would constitute an incongruent response, while choosing R+_2_ would constitute a congruent response. Evidence for outcome-specific transfer would be seen if the presence of the CS-induced a higher rate of congruent responses, as compared to incongruent responses. Consequently, outcome-specific transfer was tested considering only those trials in which a CS+_1_ or CS+_2_ was presented while choosing between R+_1_ or R+_2_ (see Table 1 for a detailed description). The rationale of the outcome-specific transfer is to test if a CS+ (i.e., a reward-paired cue) can elicit a response independently associated with the same reinforcer. To this aim, responses were categorized as congruent (choosing R+_1_ while CS+_1_ is presented or choosing R+_2_ while CS+_2_ is presented) or incongruent (choosing R+_1_ while CS+_2_ is presented or choosing R+_2_ while CS+_1_ is presented) and compared^13–15^. A transfer index was calculated, for each trial, as (number of congruent responses—number of incongruent responses) / total number of responses^13–15^. A positive score indicated a preference for congruent over incongruent responses, while a negative score the opposite. A value of zero represented the absence of outcome-specific transfer.

#### General transfer

Participants were presented with a task-irrelevant CS (either reward-associated, CS+_1_/CS+_2,_ or CS−) and required to choose between two available response options none of which was compatible with the CS currently available (i.e., the responses were always associated with a different or no outcome). So, if the CS+_1_ was presented the available response options were R+_2_ and R−; if the CS+_2_ was presented the available response options were R+_1_ and R−; if the CS− was presented the available response options could be either R+_1_ and R− or R+_2_ and R−. Evidence for general transfer would be seen if participants favored the R+ during the presentation of a CS+, as compared to the CS−. The whole task consisted of 135 randomized trials (15 repetitions for each trial type, see Table 1) and lasted for about 12 min. General transfer was tested by comparing the preference for rewarded over unrewarded responses when presented with a CS+ previously paired with a different outcome, relative to the CS− (see Table 1 for a detailed description)^13–15^. A preference index was created by subtracting, for each trial, the number of R− from the number of R+choices and dividing for the total number of responses (reward-associated responses–unrewarded responses) /total number of responses^13–15^. A positive score corresponded to a preference for R+ over R− during that trial, and vice-versa. To obtain a single indicator of transfer CS− trials were then subtracted from CS+ trials. In the resulting transfer index, a positive value indicated a preference for R+ over R− during CS+ trial, while a negative score the opposite. A value of zero represented the absence of general transfer. In the whole task, trials were equally divided into three time-points. Given the known decremental effect of responses during extinction^9,13,16^, only the first is reported in the analysis.

### Procedure

Participants seated comfortably in a silent room and their position was centered relative to the screen, at a viewing distance of about 50 cm. At the beginning of the experimental procedure, participants were instructed to play a computer game aiming to accumulate apple and orange juice to quench the thirst of a camel crossing the desert. Then the task began. In each phase of the task, the participants were required to pay attention to the screen and follow the instructions reported at the beginning of the task.

### Statistical analysis

All statistical analyses were performed using R software v3.3.2 (R Core Team, 2016) and RStudio v1.0.136 (RStudio Team, 2016). The following packages were implemented: lme4, BayesFactor, afex, stats, lsmeans, relaimpo, plotmeans, multicon. In mixed-effects models, subjects were always modeled as a random effect. All post-hoc comparisons were Bonferroni-corrected. Assumptions for the correct use of parametric statistics were always assessed. If assumptions were violated, non-parametric statistics were used instead. Results were reported by indicating, as appropriate, the statistical test value with degrees of freedom, the p-value (two-tailed), the effect size, and 95% confidence intervals. Values were reported as mean (mean) and standard deviation (sd) in the text and median and interquartile range in the plots for non-normally distributed variables. The Bayes Factor is reported for all analyses as the probability associated with the alternative hypothesis over the null hypothesis (BF_10_). The default number of Monte Carlo samples was always 10,000. The estimated proportional error (err.%) associated with the Bayes Factor is reported only of higher than 0. In all figures, data were reported using rainclouds plots^56^.

## Data Availability

Data and code used for statistical analysis are available at this link: https://osf.io/fvmzw.
